# Epigenetic silencing of glutaminase 2 in human liver and colon cancers

**DOI:** 10.1186/1471-2407-13-601

**Published:** 2013-12-14

**Authors:** Jianbin Zhang, Cheng Wang, Mingquan Chen, Jianping Cao, Ying Zhong, Liting Chen, Han-Ming Shen, Dajing Xia

**Affiliations:** 1 School of Public Health, Zhejiang University, 388 Yuhangtang Road, Hangzhou 310058, China; 2 Department of Physiology, Yong Loo Lin School of Medicine, National University of Singapore, Singapore 117597, Singapore; 3 Institute of Immunology, School of Medicine, Zhejiang University, Hangzhou 310058, China; 4 Department of Infectious Diseases, Huashan Hospital, Fudan University, Shanghai 200040, China

**Keywords:** Gls2, Methylation, Hepatocellular carcinoma, Colon cancer

## Abstract

**Background:**

Glutaminase 2 (Gls2) is a p53 target gene and is known to play an important role in energy metabolism. Gls2 has been reported to be downregulated in human hepatocellular carcinomas (HCC). However, the underlying mechanism responsible for its downregulation is still unclear. Here, we investigated Gls2 expression and its promoter methylation status in human liver and colon cancers.

**Methods:**

mRNA expression of Gls2 was determined in human liver and colon cancer cell lines and HCC tissues by real-time PCR and promoter methylation was analyzed by methylation-specific PCR (MSP) and validated by bisulfite genome sequencing (BGS). Cell growth was determined by colony formation assay and MTS assay. Statistical analysis was performed by Wilcoxon matched-pairs test or non-parametric t test.

**Results:**

First, we observed reduced Gls2 mRNA level in a selected group of liver and colon cancer cell lines and in the cancerous tissues from 20 HCC and 5 human colon cancer patients in comparison to their non-cancerous counter parts. Importantly, the lower level of Gls2 in cancer cells was closely correlated to its promoter hypermethylation; and chemical demethylation treatment with 5-aza-2′-deoxycytidine (Aza) increased Gls2 mRNA level in both liver and colon cancer cells, indicating that direct epigenetic silencing suppressed Gls2 expression by methylation. Next, we further examined this correlation in human HCC tissues, and 60% of primary liver tumor tissues had higher DNA methylation levels when compared with adjacent non-tumor tissues. Detailed methylation analysis of 23 CpG sites at a 300-bp promoter region by bisulfite genomic sequencing confirmed its methylation. Finally, we examined the biological function of Gls2 and found that restoring Gls2 expression in cancer cells significantly inhibited cancer cell growth and colony formation ability through induction of cell cycle arrest.

**Conclusions:**

We provide evidence showing that epigenetic silencing of Gls2 via promoter hypermethylation is common in human liver and colon cancers and Gls2 appears to be a functional tumor suppressor involved in the liver and colon tumorigenesis.

## Background

Hepatocellular carcinoma (HCC) is among the most common cancers worldwide, especially in Asia, with a high mortality [1]. It has been well established that hepatitis virus infection and environmental hepatocarcinogens such as aflatoxins are the main etiological factors [2,3], while the molecular pathogenesis of HCC remains largely unknown. One important underlying mechanism for HCC development is the cumulative genetic and epigenetic alterations. For instance, the epigenetic changes such as DNA methylation can lead to the inactivation of tumor suppressor genes (TSGs) that are known to play important roles in liver carcinogenesis [4,5].

DNA methylation occurs mainly at the cytosine bases of CpG island (CGI) which is found in the promoter region of genes and plays important roles in controlling gene transcription [6,7]. Once methylated, CGIs can serve as the formation of transcriptional repressive complex which subsequently leads to the transcriptional silencing of related genes [6]. CGIs are generally hypomethylated in normal cells but frequently hypermethylated in cancer cells [4]. DNA hypermethylation acts as an alternative mechanism of inactivation of tumor suppressor genes, and it is now recognized as an important mechanism during tumor initiation and progression [7,8]. In different tumor types, DNA methylation has been identified in different genes, indicating unique epigenetic changes. In HCC, DNA methylation has been described in many tumor suppressor genes, such as p53, p16^INK4a^, adenomatous polyposis coli (APC) and Ras association domain-containing protein 1 (RASSF1a) [9-11]. Since many tumor suppressor genes are silenced by DNA methylation, DNA methylation has been proposed as a marker to identify novel tumor suppressor genes [10-12].

Glutaminase 2 (Gls2) is a mitochondrial glutaminase catalyzing the hydrolysis of glutamine to glutamate and it has been identified as a p53 target gene to influence the energy metabolism [13,14]. Gls2 can regulate antioxidant defense function in cells by decreasing reactive oxygen species (ROS) levels and protect cells from oxidative stress that is known to contribute to genetic instability and cancer initiation and progression [15,16]. Compared to normal tissue, Gls2 expression is reduced in liver tumor tissues [13,14]; however, the molecular mechanism for its downregulation is still not clear.

Since DNA methylation is a common event in the silencing of tumor suppressor genes, we hypothesized that Gls2 promoter hypermethylation was responsible for its low expression in cancer cells. Our results revealed that Gls2 was downregulated in human liver and colon cancer cell lines as well as in human liver tumor tissues, and the lower level of Gls2 was correlated with its promoter hypermethylation. Moreover, overexpression of Gls2 led to cancer cell growth inhibition and cell cycle arrest. Our data thus provide useful insights into the possible role of Gls2 as a functional tumor suppressor involved in human liver and colon cancers.

## Methods

### Ethics statement

For the human normal and cancerous liver and colon tissues, written informed consent from the patients was obtained and this study was reviewed and approved by the Institutional Ethics Committee of Huashan Hospital (Shanghai, China).

### Primary tissues

For 20 HCC and 5 colon cancer patients, the tumor tissues, the adjacent non-tumor tissues and the distant normal tissues were collected from Huashan Hospital (Shanghai, China) as frozen samples. The distance between adjacent non-tumor tissue and tumor tissue boundary was 2 cm, beyond of which was regarded as distant normal tissue. The selected tumor areas had more than 80% of tumor cells as being confirmed by histology examination.

### Cell lines

Nine human liver cancer cell lines (HepG2, Hep3B, Huh7, HCC-LM3, BEL-7402, SMMC-7721, Sk-Hep1, MHCC-97H and MHCC-97 L) and six human colon cancer cell lines (HT-29, SW480, SW620, HCT116, LoVo and WiDr) were all from American Type Culture Collection (ATCC, Manassas, Va., USA). Unless specifically indicated, cells were cultured in DMEM medium (Invitrogen, Carlsbad, CA., USA) supplemented with 10% fetal bovine serum at 37°C with 5% CO2. For pharmacological demethylation, cells were treated with 5 μM 5-aza-2′-deoxycytidine (Aza) (Sigma, St. Louis, Mo., USA) for three consecutive days. Culture medium was changed every 24 hours. An equivalent concentration of vehicle (DMSO) was used as the control.

### RNA/DNA extraction, reverse transcription and real-time PCR

Total RNA and genomic DNA from both cultured cells and human tissue samples were extracted using Trizol reagent (Invitrogen) according to the manufacturer’s instructions and their concentrations were quantified by NanoDrop 1000 (Wilmington, DE., USA). A reverse transcription reaction was performed using 1 μg of total RNA with High Capacity cDNA Reverse Transcription kit (Applied Biosystems, Foster City, CA, USA). The mRNA level of Gls2 was determined by real-time PCR using SYBR Green Master Mix Kit and ABI 7500 Real-Time PCR System (Applied Biosystems, Foster City, CA, USA). Glyceraldehyde-3- phosphate dehydrogenase (GAPDH) was used as an internal control. The 2^-△△CT^ method was used to analyze the relative changes in Gls2 expression from real-time PCR experiments [17]. Real-time PCR was performed in triplicate. Primers used for Gls2 were: Gls2-F 5′-TCCAGCTGT -GTTCTGTGGAG-3′ and Gls2-R 5′-GCAAACTGGCCAGAGAA GTC-3′ (175 bp product).

### Bisulfite treatment of DNA and methylation-specific PCR

Genomic DNA was treated with bisulfite using Zymo DNA Modification Kit (Zymo Research, Orange, CA, USA) according to the protocol provided. Methylation -specific PCR (MSP) was carried out for 40 cycles with annealing temperature at 60°C. Methylation-specific primers were: Gls2-MF 5′-GTTGGTCGGTTGTTTTTGTTC-3′ and Gls2-MR 5′-AAAACTACGAATAATAAAATTATCGTA-3′, and unmethylation -specific primers were: Gls2-UF 5′-TTTGTAGTTTTATGGTGTTTTTGG-3′ and Gls2-UR 5′-CCTTACAACAATCTACAAATACTTCCAC-3′.

### Bisulfite genome sequencing

Bisulfite-treated genomic DNA was amplified using bisulfite genome sequencing (BGS) primers, Gls2-BF: 5′-GGGTTTATTTTTATTTAGTTTTTTTT-3′ and Gls2-BR: 5′-AAATAAACAATACCCAAATCCAATC-3′. PCR products were purified with Illustra GFX^TM^PCR and gel band purification kit (GE Healthcare Life Science, Uppsala, Sweden) and cloned into pCR4-TOPO vector for sequencing (Invitrogen). At least four colonies were randomly chosen for plasmid extraction and sequencing analysis using the ABI PRISM Big Dye Terminator Cycle Sequencing Kit in the ABI 3100 sequencer (Applied Biosystems).

### Cell growth and colony formation assay

Cells growth was determined by using the MTS assay (Promega, Madison, WI, USA). Briefly, cells transfected with either empty vector or 3xFlag-tagged Gls2 expressing vector were cultured in a 96-well plate in complete DMEM for 24 hours. The quantity of formazan was measured at 490 nm absorbance after one hour incubation with CellTiter 96A Queous One Solution Reagent following instructions provided. After overexpression of Gls2, human liver cancer cell SMMC-7721 and colon cancer cell HCT116 was used for the colony formation assay. Cells were cultured overnight in a 12-well plate (5 × 10^5^ cells per well) and transfected with either empty vector or 3 × Flag-tagged Gls2 expressing vector [7] (kindly provided by Dr. Tomoaki Tanaka, Chiba University, Japan) using FuGENE 6 (Roche Applied Science, Mannheim, Germany). Forty-eight hours later, the transfectants were replated in 6-well plate and cultured for 10 ~ 15 days in complete DMEM medium containing G418 (400 μg/ml). Surviving colonies were stained with Gentian Violet after methanol fixation and visible colonies were counted. The experiments were repeated three times.

### Cell cycle analysis

SMMC-7721 and HCT116 cells transfected with either empty vector or 3 x Flag-tagged Gls2 expressing vector were trypsinized, washed in PBS and fixed in ice-cold 70% ethanol in PBS. After washing, the fixed cells were treated with 0.01% RNase (10 mg/ml, Sigma, St. Louis, MO, USA) for 10 minutes at 37°C and then stained with 0.05% propidium iodide for 20 minutes at 4°C in dark. The cell cycle distribution was determined using a FACScanto flow cytometry (Becton Dickinson, Mountain View, CA, USA) and analyzed with Modfit software (Phoenix, San Diego, CA, USA).

### Western blotting

The transfected cells with either empty vector or 3 x Flag-tagged Gls2 expressing vector were lysed in M2 lysis buffer (20 mM Tris at pH 7, 0.5% NP-40, 250 mM NaCl, 3 mM EDTA, 3 mM EGTA, 2 mM dithiothreitol, 0.5 mM phenylmethylsulfonyl fluoride, 20 mM glycerol phosphate, 1 mM sodium vanadate and proteinase inhibitor cocktail). Equal amount of protein was fractionated on SDS-PAGE and transferred onto PVDF membrane (Bio-Rad, CA, USA). After blocked with 5% nonfat milk, the membrane was probed with designated first and second antibodies (Cell Signaling Technology, Beverly, MA, USA) and developed with enhanced chemiluminescence method and visualized by Kodak Image Station 440CF (Kodak, USA). The band density was quantified using image processing program and normalized to that of the control group. The first antibodies used in this study included the following: Phospho-cdc25c (ser216) from Cell Signaling, #4901, 1:1000 dilution, p21 Waf1/Cip1 (12D1) from Cell Signaling, #2947, 1:1000 dilution, Cyclin D1 (DCS6) from Cell Signaling, #2926, 1:2000 dilution, and Anti-FLAG® antibody from Sigma, #F7425, 1:10000 dilution.

### Statistical analysis

The numeric data were expressed as mean ± SD. The difference in Gls2 mRNA levels between paired tissue samples was determined by the Wilcoxon matched-pairs test. The difference in Gls2 mRNA levels between two groups was determined by the non-parametric t test. *P* < 0.05 was taken as statistical significance. All the tests were performed by Graphpad Prism 5.0.

## Results

### Downregulation of Gls2 in human liver and colon cancer

**Figure 1:**
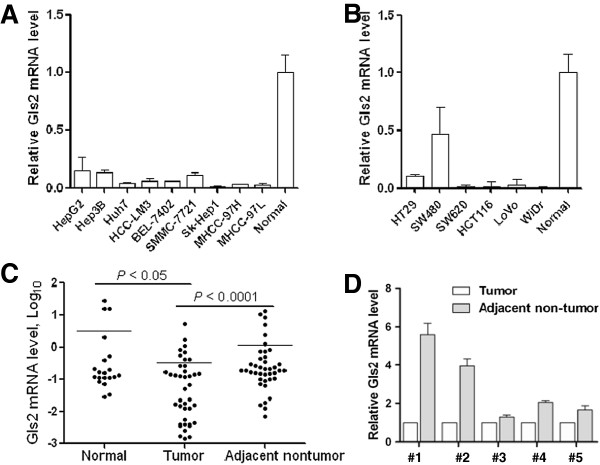
**Downregulation of Gls2 mRNA level in human liver and colon cancer cells and HCC tissues.** Gls2 mRNA level in human liver **(A)** and colon **(B)** cancer cells and normal tissues was determined by real-time PCR. GAPDH was used to normalize the template amount. **(C)** Gls2 mRNA level in human liver tumor tissues (n = 40), adjacent non-tumor tissues (n = 40) and normal tissues (n = 20) was determined by real-time PCR. The lines denote the mean value. **(D)** Gls2 mRNA level in human colon tumor tissues and adjacent non-tumor tissues (n = 5) was also determined by real-time PCR.

In this part of our study, we first examined Gls2 mRNA level in an array of human liver and colon cancer cell lines. Using real-time PCR, reduced level of Gls2 mRNA was found in a group of human liver cancer cells (Figure 1A) and colon cancer cells (Figure 1B) when compared to the human normal liver and colon tissues, respectively. More importantly, we found that Gls2 expression was significantly downregulated in 85% of human liver tumor tissues when compared to the adjacent non-tumor tissues and distant normal tissues (Figure 1C). Similar trend was also observed in colon cancer tissues when we compared the Gls2 mRNA level among 5 pairs of colon tumor tissues vs the adjacent non-tumor tissues (Figure 1D). Such observations are generally consistent with the earlier reports that Gls2 is downregulated in cancers [13,14].

### Demethylation treatment increased Gls2 expression in human liver and colon cancer cells

**Figure 2:**
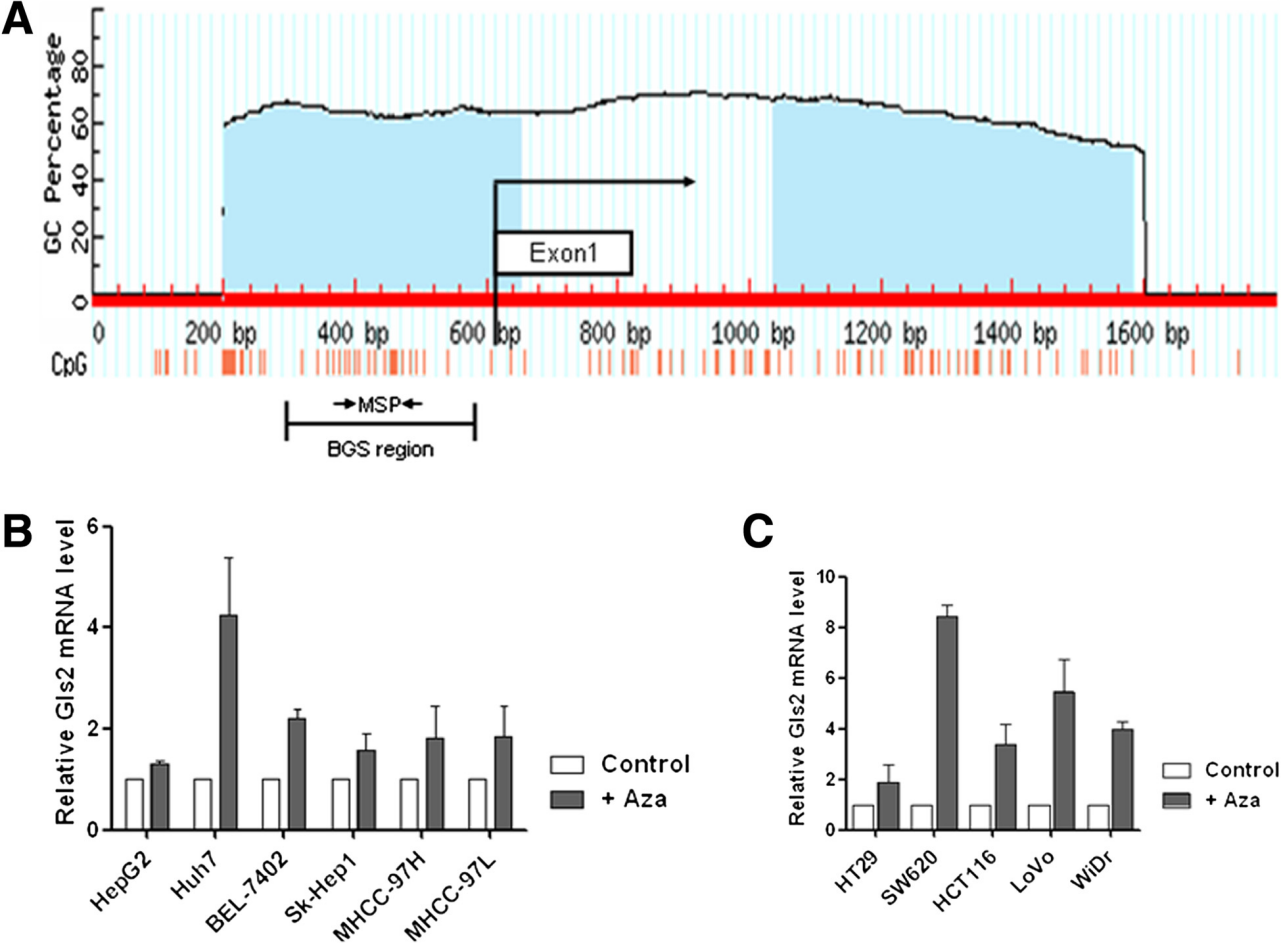
**Pharmacological genome demethylation upregulated Gls2 expression in human liver and colon cancer cells. (A)** Schematic structure of the Gls2 CpG island (CGI), with the exon 1 and MSP and BGS regions indicated. Each short vertical line represents one CG site. The position of MSP and BGS regions was marked with arrows. MSP, methylation- specific PCR; BGS, bisulfite genome sequencing. Gls2 expression in human liver **(B)** and colon **(C)** cancer cells after demethylation treatment with 5 μM Aza for three days. Data was presented as mean ± SD from three independent measurements.

Using CpG island searcher, two typical CGIs were found around Gls2 exon 1 using the following criteria: GC content > 55%, Observed CpG/Expected CpG > 0.65, and length > 500 bp (Figure 2A), suggesting that DNA methylation may occur in the promoter region of Gls2. In order to know whether Gls2 expression is correlated to DNA methylation, both liver and colon cancer cells were treated with a demethylation reagent Aza. As shown in Figure 2B, such a treatment markedly enhanced Gls2 mRNA level in almost all the selected human liver cancer cells. Similar results were also found in the five human colon cancer cells tested (Figure 2C). Therefore, such data indicate that the downregulaton of Gls2 expression in human liver and colon cancer cells is mediated by its promoter hypermethylation.

### Promoter hypermethylation of Gls2 in human liver and colon cancer cells and HCC tissues

**Figure 3:**
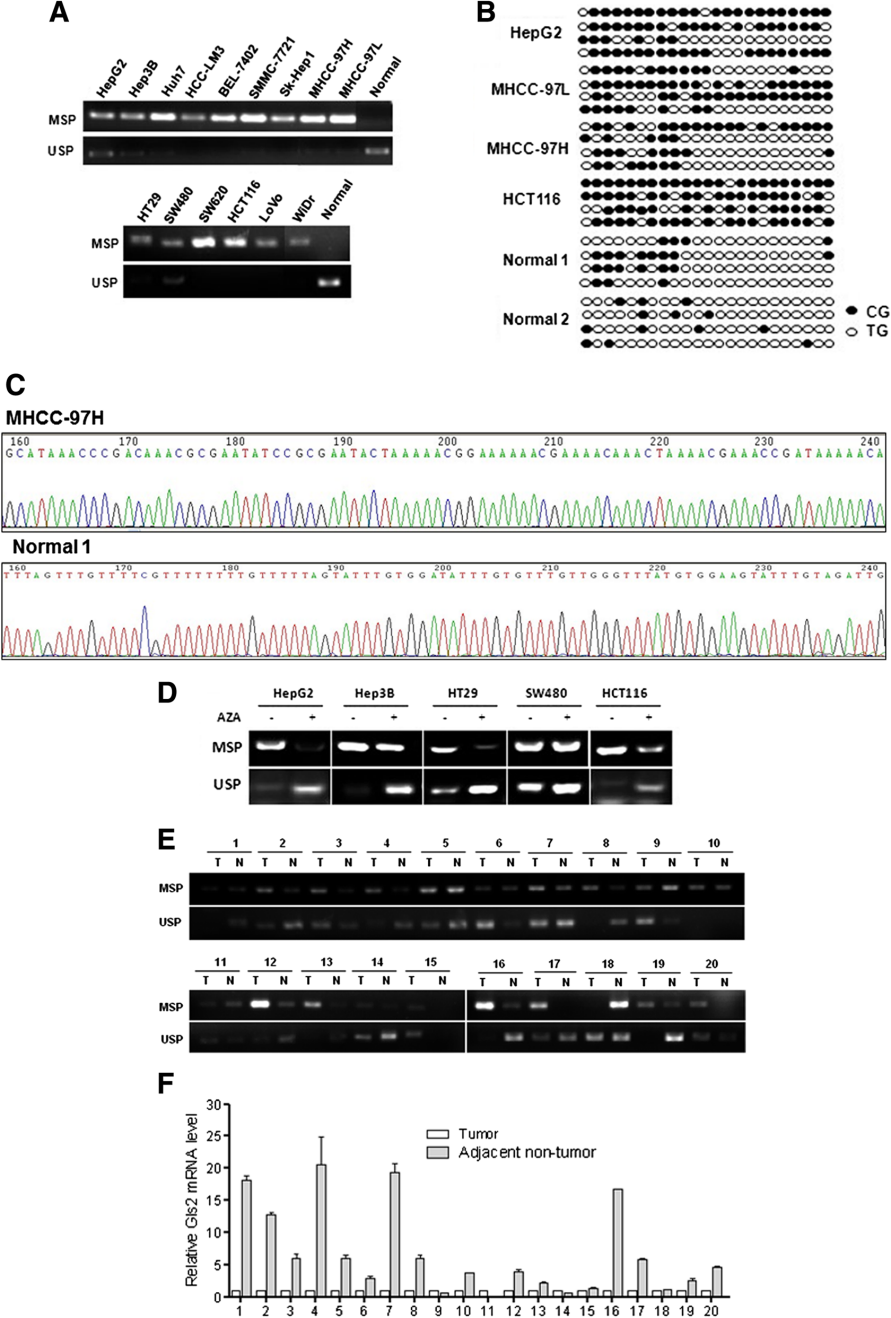
**Gls2 promoter region was hypermethylated in human liver and colon cancers.** The Gls2 promoter methylation status in cancer cells was analyzed by MSP **(A)** and BGS **(B-C)**. For BGS, each circle indicates one CG site and circles filled in black represent methylated CG sites. One row of circles represents a single colony **(B)** and a chromatogram representative of BGS results in MHCC-97H and normal liver tissue was also shown **(C)**. USP, unmethylation-specific PCR; Normal 1, normal liver tissue; Normal 2, normal colon tissue. **(D)** Gls2 promoter methylation status in cancer cells after demethylation treatment. **(E)** The promoter methylation status of Gls2 in 20 pairs of human HCC tissues and adjacent non-tumor tissues was determined by MSP. **(F)** Gls2 mRNA level in these tissues was measured by real-time PCR. GAPDH was used to normalize the template amount. USP, unmethylation-specific PCR. “T” indicates tumor tissues and “N” represents adjacent non-tumor tissues.

In order to confirm the above findings, we then directly determined the promoter methylation status of Gls2 in both human liver and colon cancer cells by using methylation-specific PCR (MSP). As shown in Figure 3A, Gls2 promoter methylation was readily detected in human liver and colon cancer cells but not in the normal tissues. Bisulfite genomic sequencing (BGS) also revealed that Gls2 promoter was heavily methylated in HepG2, MHHC-97H, MHCC-97 L and HCT116 cells, while it was weakly methylated in human normal liver and colon tissue (Figure 3B). Representative chromatograms of MHCC-97H cancer cells and normal liver tissue were also presented (Figure 3C). Moreover, after demethylation treatment with Aza, lower methylation level of Gls2 was detected in HepG2, Hep3B, HT29 and HCT116 cells, but not in SW480 cells (Figure 3D).

To further confirm the pathological relevance of Gls2 promoter hypermethylation in mediating its downregulation in tumorigenesis, we further analyzed the Gls2 promoter methylation in 20 pairs of human primary HCC tissues and adjacent non-tumor tissues. As shown in Figure 3E, higher methylation level of Gls2 was detected in 60% of human liver tumor tissues using MSP. Importantly, such a pattern is found to be generally consistent with the relative mRNA level of Gls2 measured in the group of 20 pairs of human primary HCC tissues and adjacent non-tumor tissues (Figure 3F). In addition, we also attempted to summarize and compare the clinical-pathological characteristics of the 20 liver cancer patients in terms of Gls2 promoter methylation status (Table 1). It appears that liver cancers with Gls2 promoter methylation were found to have higher percentage of abnormal AFP and higher TNM stage, although no statistical differences were found, probably due to the relative small sample size. Taken together, data from this part of our study indicate that hypermethylation-mediated downregulation of Gls2 is pathologically relevant in the tumorigenesis of HCC.

**Table 1:** Clinical-pathological features of the 20 liver cancer patients

**Characteristics**	**Gls2-methylated**	**Gls2-unmethylated**
Age, years	51 ± 10.6	53 ± 12.9
(n)	(13)	(7)
Gender		
M	11 (84.6%)	6 (85.7%)
F	2 (15.4)	1 (14.3%)
HBV infection		
Positive	10 (76.9%)	5 (71.4%)
Negative	3 (23.1%)	2 (28.6%)
AFP level		
Normal	4 (30.8%)	3 (42.9%)
Abnormal	9 (69.2%)	4 (57.1%)
TNM stage		
I-II	3 (23.1%)	2 (28.6%)
III-IV	10 (76.9%)	5 (71.4%)

### Ectopic expression of Gls2 reduced cancer cell growth

**Figure 4:**
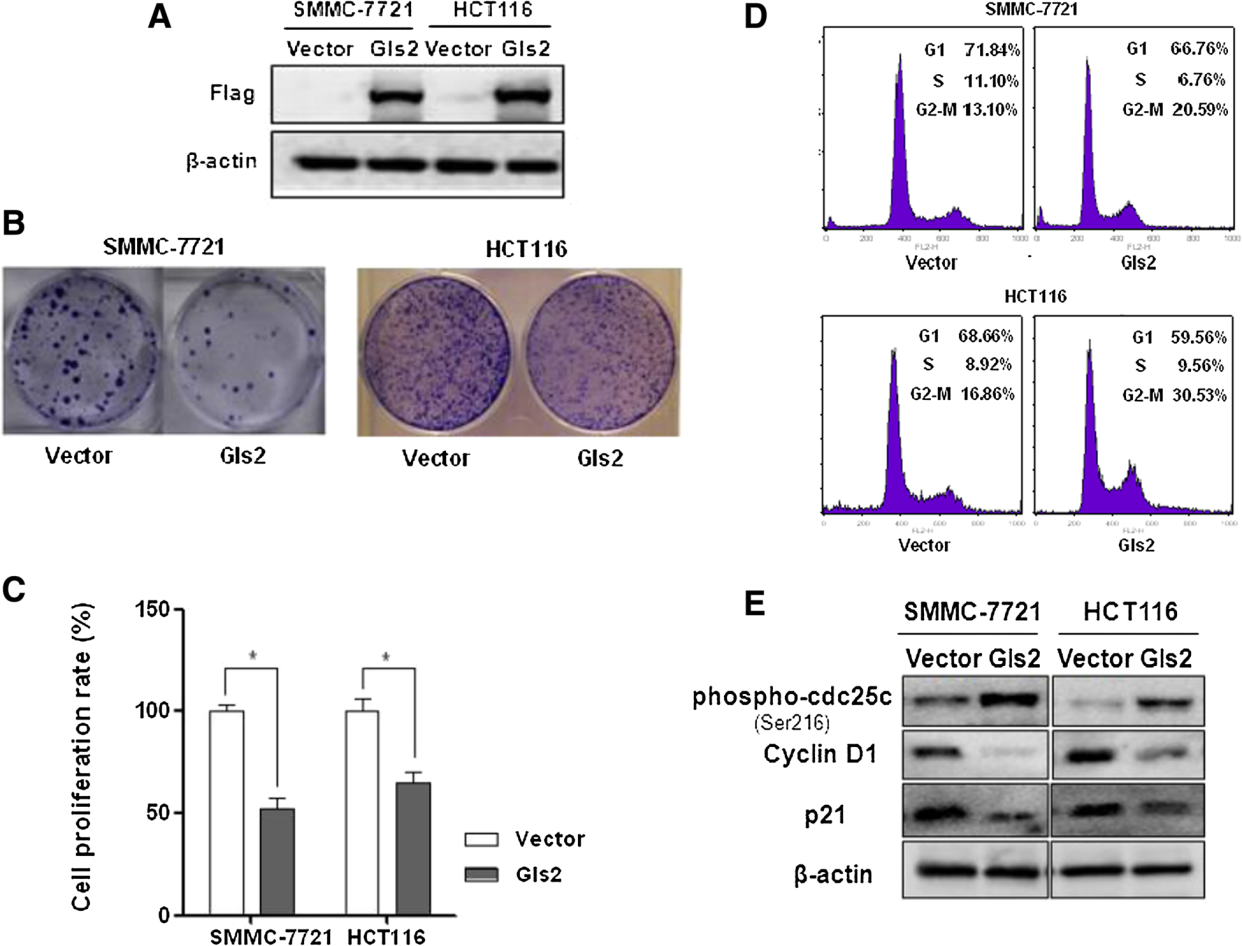
**Gls2 suppressed cancer cell growth and induced cell cycle arrest. (A)** Ectopic expression of Gls2 in human liver and colon cancer cells. SMMC-7721 and HCT116 cells were transiently transfected with either empty vector or 3 × Flag-tagged Gls2 expressing vector and the expression level of Gls2 was measured by western blot. β-actin served as a loading control. **(B)** Cell proliferation ability was measured by colony formation assay, which was performed as described in Methods. **(C)** Overexpression of Gls2 inhibited cancer cell proliferation measured by MTS assay. The values obtained from transfected and control cells represent mean ± SD of three independent experiments. *p < 0.05 between the two groups were calculated using individual Student’s t-test. **(D)** Changes of the cell cycle profiles in cancer cells after Gls2 overexpression were determined using flow cytometry, with representative histograms shown for each treatment. **(E)** Changes of cell cycle regulators (phospho-cdc25 (Ser216), p21 and cyclin D1) were determined using western blotting. β-actin was used as the internal control.

To further investigate the potential tumor suppressive role of Gls2, human liver cancer cells SMMC-7721 and human colon cancer cells HCT116 were transfected with either empty vector or 3 × Flag-tagged Gls2 expressing vector, and the Gls2 expression in SMMC-7721 and HCT116 cells was confirmed by western blotting (Figure 4A). Ectopic expression of Gls2 in both cells markedly reduced the number of cell colony formed under the selection of G418 (Figure 4B). When compared to cells transfected with empty vector, overexpression of Gls2 significantly decreased cell growth rate measured by MTS (*p* < 0.05, Figure 4C). These data support the tumor suppressive function of Gls2.

To determine the molecular mechanism by which Gls2 suppressed cancer cell proliferation and colony formation, we further investigated the effect of Gls2 overexpression on cell cycle. Gls2 overexpression led to a significant increase in the number of cells in G2/M phase in both SMMC-7721 and HCT116 cells (Figure 4D). To understand the underlying mechanism for Gls2-mediated G2/M arrest, we examined some of the key cell cycle regulators changes. Overexpression of Gls2 in cancer cells induced phosphorylation of the protein phosphatase cdc25c, a key regulator in the G2/M checkpoint, together with a significant reduction in p21 and cyclin D1 (Figure 4E).

## Discussion

In this study, we first provided the novel evidence linking the lower level of Gls2 with its promoter hypermethylation in both human liver and cancer cells, with the following observations: (i) Gls2 was downregulated in human liver and colon cancer cells (Figure 1A and B), and in primary hepatocellular carcinomas tissues in comparison to both the distant normal and adjacent non-tumor tissues (Figure 1C); (ii) demethylation treatment with Aza markedly upregulated Gls2 expression in human liver and colon cancer cells (Figure 2B and C); (iii) higher DNA methylation levels were found in human liver and colon cancer cells and in HCC samples; and such changes were well correlated to the reduced Gls2 mRNA level in both cancer cells and cancer tissues (Figure 3A-F). Moreover, overexpression of Gls2 led to cell growth inhibition and cell cycle arrest (Figure 4). Thus, our findings demonstrate that epigenetic silencing of Gls2 via promoter hypermethylation is an important event relevant to liver and colon tumorigenesis.

DNA methylation has been well established as an important molecular mechanism underlying tumorigenesis [8,11]. Measuring DNA methylation not only serves as a marker to identify novel TSGs but also can be used as a sensitive marker for cancer diagnosis and prognosis [5,18]. In chronic hepatitis C patients, methylation frequencies in many TSGs, such as RASSF1, CDKN2A, APC, and RUNX3, were associated with shorter time-to-HCC occurrence [19]. Therefore, epigenetic inactivation of these genes in chronic hepatitis C patients provides a prognostic marker for determining the risk for developing HCC. Besides, poor prognosis of human hepatocellular carcinoma has been also found to be associated with epigenetic silencing of some TSGs [20]. Here, we determined Gls2 methylation status in 20 pairs of primary hepatocellular carcinomas (Figure 3E-F), suggesting that Gls2 downregulation is attributed to its promoter hypermethylation. It is thus believed that detection of Gls2 promoter methylation in liver tissues could be a useful biomarker for diagnosis and prognosis of liver cancer.

Although promoter methylation is an important mechanism in silencing Gls2 in human liver and colon cancer cells, we cannot exclude the involvement of other mechanisms responsible for Gls2 downregulation, such as histone acetylation [21]. For instance, in Hep3B, HCC-LM3, SMMC-7721 and SW480 cells, Gls2 expression failed to be fully upregulated by Aza treatment. Insteadly, combined treatment with Aza and histone deacetylase inhibitor TSA could increase Gls2 expression in these cancer cells (data not shown), suggesting that histone modification may also mediate Gls2 downregulation in cancer cells.

In our study, we found that overexpression of Gls2 markedly suppressed cancer cells proliferation and colony formation. It has been reported that Gls2 can exert its tumor suppressive function via reduction of oxidative stress-induced DNA damage and mutations [15]. However, other molecular mechanisms responsible for Gls2 as a tumor suppressor remain unknown. It has been well-established that cell cycle checkpoints are important control mechanisms in maintaining tissue homeostasis and one of the checkpoints, G2/M checkpoint blocks the entry into mitosis when DNA is damaged [22]. The p53 protein can regulate G2/M transition either through the induction of p21 and stratifin, a protein that normally sequesters cyclin B1-cdc2 complexes in the cytoplasm [23,24], or through the induction of apoptosis [25,26]. Since Gls2 has been identified as a p53 target gene, we determined the effect of Gls2 on cell cycle progression and found that Gls2 overexpression induced G2/M phase cell cycle arrest (Figure 4C and D), indicating its important role in tumor suppression.

In addition to the discussions above, we acknowledge that there are a number of limitations in this study. Firstly, due to the relative small samples size, we are unable to determine the association of Gls2 promoter methylation status with the clinical-pathological characteristics (Table 1). Secondly, we did not perform the analysis of p53 in the human liver cancer tissues and thus we are unable to link p53 activity with Gls2 expression level in those patients. And finally, since the adjacent non-cancerous tissues were not obtained via microdissection, technically we could not exclude the presence of cancerous cells in those specimens, which may explain the presence of MSP in some of the non-cancerous tissues as shown in Figure 3E.

## Conclusions

In summary, we found that Gls2 is frequently downregulated in human liver and colon cancer cells and also in primary HCC tissues, and such downregulation is correlated to its promoter hypermethylation. Moreover, ectopic expression of Gls2 suppressed cancer cell growth through induction of cell cycle arrest. Therefore, it appears that Gls2 is an important tumor suppressor involved in the liver and colon tumorigenesis. Our results also suggest that Gls2 is a valuable molecular target for the detection and treatment of these malignancies.

## Competing interests

The authors who have taken part in this study declared that they do not have anything to disclose regarding funding or conflict of interest with respect to this manuscript.

## Authors’ contributions

The study was conceived by JZ and DX. Experiments were carried out by JZ, CW, MC, JC, YZ and LC. Statistical analysis was carried out by JZ. Manuscript was written by JZ, HMS and DX. All authors read and approved the final manuscript.

## Pre-publication history

The pre-publication history for this paper can be accessed here:

http://www.biomedcentral.com/1471-2407/13/601/prepub
